# Therapeutic components of digital counseling for chronic heart failure

**DOI:** 10.3389/fpsyt.2022.888524

**Published:** 2022-10-20

**Authors:** Gabriel C. Fezza, Stephanie Sansone, Robert P. Nolan

**Affiliations:** ^1^Behavioral Cardiology Research Unit, University Health Network (UHN), Toronto, ON, Canada; ^2^Faculty of Health, York University, Toronto, ON, Canada; ^3^Faculty of Medicine, University of Toronto, Toronto, ON, Canada; ^4^Peter Munk Cardiac Centre, Toronto General Hospital Research Institute, University Health Network, Toronto, ON, Canada; ^5^Ted Rogers Centre for Heart Research, Faculty of Medicine, University of Toronto, Toronto, ON, Canada

**Keywords:** cognitive behavioral therapy, digital health, eHealth, heart failure, motivational interviewing, quality of life, self-care, telemedicine

## Abstract

**Background:**

Task force statements support the use of cognitive behavioral therapy (CBT) and motivational interviewing (MI) to promote self-care in chronic heart failure (CHF) patients. Digital counseling interventions have the potential to complement conventional programs. However, therapeutic components of digital programs associated with improved outcomes are not clearly established.

**Objective:**

Identify therapeutic components of the Canadian e-Platform to Promote Behavioral Self-Management in Chronic Heart Failure (CHF-CePPORT) protocol that were associated with improved health-related quality of life (HRQL).

**Materials and methods:**

Ordinal logistic regression was used to identify therapeutic components of the CHF-CePPORT protocol. The primary outcome was the 12-month Kansas City Cardiomyopathy Questionnaire Overall Summary (KCCQ-OS) tertile. Logistic regressions determined the association between 12-month KCCQ-OS tertile, using logon hours for key segments of the protocol, modality of content delivery, and clinical themes.

**Results:**

A total of 117 patients were enrolled in the e-Counseling arm of the CHF-CePPORT trial. Median age was 60 years (IQR 52–69). Total logon hours in the initial 4-month segment of CHF-CePPORT (Sessions 1–16) was associated with increased 12-month KCCQ-OS tertile (Odds Ratio, OR = 1.31, 95% CI, 1.1–1.5, *P* = 0.001). Within sessions 1–16, improved KCCQ-OS was associated with logon hours for self-assessment tools/trackers (OR = 1.49, 95% CI, 1.1–2.0, *P* = 0.007), and videos (OR = 1.57, 95% CI, 1.03–2.4, *P* = 0.04), but not for CHF information pages.

**Conclusion:**

This study highlights the importance of using evidence-based guidelines from CBT and MI as core components of digital counseling, delivered through videos and interactive tools/trackers, to improve HRQL with CHF.

## Introduction

International task force statements from professional cardiovascular health societies emphasize the importance of patient self-care in the management of chronic heart failure (CHF) (1, 2). Engagement in self-care behaviors has been shown to have a positive effect on health-related quality of life (HRQL) and to reduce rates of CHF-related mortality and hospitalization (3). These task force statements endorse the use of behavioral counseling to improve CHF self-care and health status (1, 2). Protocols of behavioral counseling that promote CHF self-care have not been clearly established. However, key components of conventional face-to-face programs are consistent with well-established evidence-based models of counseling that include cognitive behavioral therapy (CBT) and motivational interviewing (MI) (1, 2, 4, 5).

In this period of the COVID-19 pandemic, the maintenance, monitoring, and management of self-care behaviors among patients with CHF has become increasingly important to reduce hospital readmissions and to maintain health status (2). Digital health interventions have become increasingly prominent (2, 6), and utilized to support patient CHF self-care (7). These digital interventions have the potential to complement conventional clinic-based treatment programs for CHF patients in a manner that is efficacious, accessible, and replicable (6, 8).

Digitally based counseling programs have been reported to improve HRQL in patients with CHF. Different modalities of digitally based counseling programs include telemonitoring, video monitoring, and home telehealth. To our knowledge, a detailed analysis has not been conducted of therapeutic benefit associated with individual components within these programs (9). Patient engagement in logging onto these programs is in the range of 55–62% (10, 11), which is similar to engagement rates for completing sessions in conventional behavioral programs (12). This moderate level of patient engagement highlights the need to specify therapeutic components of digital counseling for CHF patients. This in turn will increase the likelihood of improving the replicability and standardization of these programs. A recent policy paper for digital health highlights effective features of patient-centered models of care (13). Examples of effective features include goal setting, having a concrete behavioral goal for change, and an ability to monitor your progress. While those guidelines advocate the use of evidence-based models of counseling, they do not specify how these features are integrated into these models of digital counseling (14). The present study was undertaken to identify therapeutic components of an automated digital counseling program that were associated with improved HRQL in the Canadian e-Platform to Promote Behavioral Self-Management in Chronic Heart Failure trial (CHF-CePPORT: ClinicalTrials.gov NCT01864369) (15).

## Materials and methods

### Participants

This study focused on patients who were randomized to the e-Counseling + Usual Care intervention arm of the CHF-CePPORT trial. Patient recruitment began in January 2014 and the final 12-month assessment was completed in February 2018 (15). Informed written consent was given by participants during enrolment to participate in the trial. Inclusion criteria for CHF-CePPORT consisted of patients ≥ 18 years of age, with New York Heart Association (NYHA) class I–III and left ventricular ejection fraction (LVEF) ≤ 45. Patients were required to be stable for 12 months prior to enrolment with no worsening of CHF for 1 month prior to enrolment, as determined by the referring cardiologist.

Patients were included if they were not currently enrolled in a formal exercise program, had comprehension of English or French, and provided informed written consent. Patients were excluded if they had current symptomatic hypotension, persistent systolic or diastolic hypertension, or clinically significant comorbidities (e.g., cancer, chronic kidney failure). Patients were also excluded if they were diagnosed with a major psychiatric disorder (e.g., psychosis).

### Study design

This investigation was a sub-study of CHF-CePPORT, which has been reported previously for both the protocol and primary outcome (15, 16). Briefly, this trial was a phase two, multi-center randomized controlled trial with a two-parallel group, double blind design, and with repeated assessments at baseline, 4- and 12-months (15, 16). CHF-CePPORT was designed to evaluate the efficacy of an evidence-based and clinically organized e-counseling protocol that promoted adherence to recommended guidelines for exercise, diet, prescribed medications, and smoke-free living over a 12-month period. Eligible CHF patients were recruited across three Canadian sites: University Health Network (Toronto), Providence Health Care (Vancouver), and the Ottawa Heart Institute. Patient recruitment was voluntary and the content of CHF-CePPORT was complementary to usual care. It was introduced to participants as a as a research study. All participants were randomly assigned to either e-Counseling + Usual Care or e-Info Control + Usual Care. The primary endpoint of CHF-CePPORT was 12-month quality of life as assessed by the Kansas City Cardiomyopathy Questionnaire Overall Summary (KCCQ-OS) (17).

### Study interventions and assessments

The automated counseling protocol for the e-Counseling arm of CHF-CePPORT has been previously described (15, 16). Briefly, the protocol was organized by 28 sessions that were sent to patients proactively *via* an email that contained a URL to the webpages for each randomized group. Emails were sent to patients weekly for months 1–4, bi-weekly for months 5–8, and monthly for months 9–12. The Control intervention included session content that was based on an amalgamation of publicly available educational information on guidelines for self-managing CHF from the Canadian Heart Failure Association, American Heart Association, and the European Society of Cardiology. The Control and e-Counseling sessions included information aimed at improving self-help skills for adhering to recommended self-care behaviors for medications, exercise, fruit and vegetable intake, restriction of sodium and fluids, and smoke-free living. The e-Counseling intervention promoted adherence to self-care behaviors by utilizing core components of MI and CBT using different digital modes of presentation: information pages (comprised of narrative script with illustrations), interactive self-assessment tools/trackers, and videos (expert guidelines for self-care, dramatic vignettes, and peer discussion). For the e-Counseling sessions, key features from MI helped patients build their readiness for change through validating their stage of readiness and guiding them to identify goals for lifestyle change that were connected to their priorities for living well. In addition, core components of CBT provided a step-by-step guide to plan and initiate self-care behavior change, and patient efficacy was reinforced using performance-based feedback through interactive self-monitoring tools (e.g., self-assessment forms and interactive trackers) (18).

### Health-related quality of life outcomes

Health-related quality of life was assessed using the KCCQ-OS score at baseline, 4-, and 12-months. The KCCQ-OS incorporated patient reported symptoms of CHF, physical limitations, social function, and quality of life (17).

### Statistical analysis

Mann–Whitney *U* tests were used to analyze continuous background variables, and Chi Square tests were used for categorical variables. The 12-month KCCQ-OS scores were transformed into tertiles (range, ≤ 74, 74.1–90.4, and 90.5–100) due to the severe skewness of scores and a clinically meaningful ceiling effect at baseline as detailed in the CHF-CePPORT primary outcome paper (15). The tertile ranges reflect fair, good, and excellent health status respectively. Scores in the higher range (two upper tertiles) of the KCCQ are well-established to predict decreased levels of morbidity and mortality in the CHF population (19).

**FIGURE 1:**
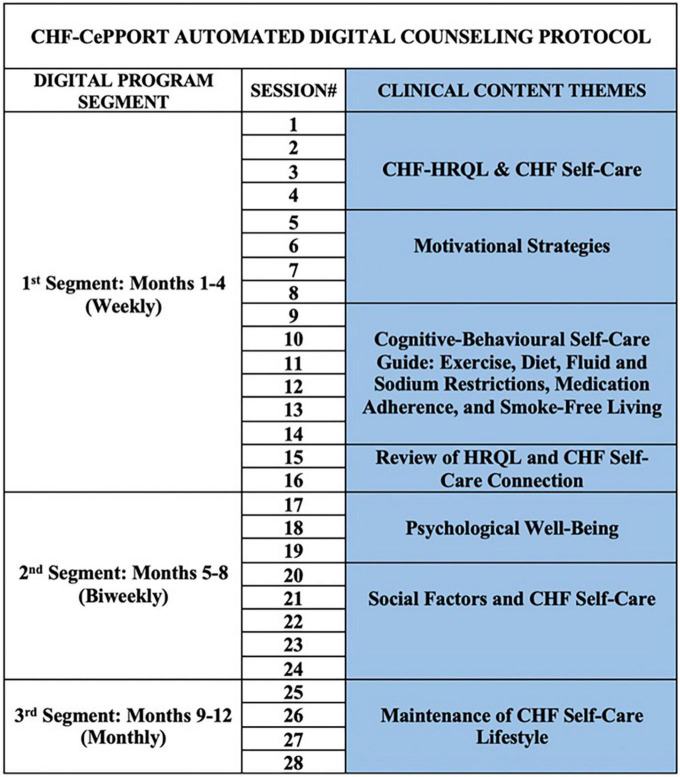
CHF-CePPORT digital counseling protocol. CHF, chronic heart failure; HRQL, health-related quality of life.

Total patient logon time (hours) was used in ordinal logistic regression analyses to determine the components of the protocol that were associated with higher KCCQ-OS tertile at 12 months. These components included: program segment over 12 months, modality of content accessed by patients, and clinical content themes in logon sessions (Figure 1). All analyses were controlled for baseline KCCQ-OS tertiles, age, LVEF (< 35, 35–40, 41–45).

The analyses were planned in a successive order according to the following objectives to identify the components of the program that were positively associated with higher KCCQ-OS tertile scores:

1. the association between 12-month KCCQ-OS tertile and logon time (hours) for the three successive periods of the CHF-CePPORT protocol: Baseline to 4 months, 4–8 months, and 8–12-months. These three time periods were characterized by proactive contact with patients on a weekly, biweekly, and monthly schedule, respectively.
2. the association between logon time for mode of content accessed by patients (information, video, or interactive tools/trackers) in automated digital counseling sessions and 12-month KCCQ-OS tertile.
3. the association between logon time for clinical themes such as quality of life, self-care behavior, and social functioning presented by digital counseling sessions and 12-month KCCQ-OS tertile.

## Results

### Patient characteristics

Chronic heart failure patients (*n* = 117) in this study received the e-Counseling intervention in the CHF-CePPORT trial. Median age was 60 years (IQR 52–69), who received the patient-centered e-counseling protocol (Table 1).

**TABLE 1:** Background characteristics of e-Counseling + usual care arm of CHF-CePPORT trial (*n* = 117).

Characteristics	*n*, median	%, (IQR)
Declared gender, female	24	20.5
Age, years	60.0	(52–69)
Education:		
≤ Secondary	33	28.2
Post-secondary	84	71.8
LVEF%:		
< 35	57	48.7
35–40	31	26.5
41–45	29	24.8
NYHA functional class:		
1	45	41.3
2	48	44.0
3	16	14.7

LVEF, left ventricular ejection fraction; NYHA, New York Heart Association.

### Analysis 1: Association between CHF-CePPORT program segment and 12-month KCCQ-OS

Total logon time (in hours) on the CHF-CePPORT platform during the initial 4 months of the program (sessions 1–16), when patient access to new digital sessions was scheduled weekly, was positively associated with the 12-month KCCQ-OS tertile (*P* = 0.001). Total logon time (in hours) was not associated with 12-month KCCQ-OS tertile for the second and third segments of the program, when access to new digital sessions was scheduled biweekly (sessions 17–24), and monthly (sessions 25–28), respectively (Table 2 and Supplementary Table 1 for details).

**TABLE 2:** Association between logon hours for CHF-CePPORT program segments and 12-month KCCQ-OS tertile.

Segment of program	Session	Schedule of contact	OR	95% CI	*P*-value
Month 1–4	Sessions 1–16	Weekly	1.31	(1.1–1.5)	0.001
Month 5–8	Sessions 17–24	Biweekly	1.26	(0.9–1.7)	0.13
Month 9–12	Sessions 25–28	Monthly	1.42	(0.8–2.7)	0.28

Each OR, odds ratio corresponds to a separate ordinal logistic regression analysis.

### Analysis 2: Modality of digital counseling (information, videos, and tools/trackers) and 12-month KCCQ-OS

The association between digital counseling modality and 12-month KCCQ-OS was limited to the initial program segment (sessions 1–16), given the finding noted immediately above. The modality of content delivery that was associated with higher 12-month KCCQ-OS tertile were videos (*P* = 0.04) and interactive tools/trackers (*P* = 0.007). Patient logon time for digital pages that provided educational information on CHF was not associated with higher 12-month KCCQ-OS tertile (Table 3 and Supplementary Table 2 for details).

**TABLE 3:** Association between logon time for digital counseling modalities in sessions 1–16 and 12-month KCCQ-OS tertile.

Modality of content delivery	OR	95% CI	*P*-value
Information/education pages	1.01	(0.5–2.2)	0.80
Videos: Expert guideline, dramatic vignettes, and peer discussion	1.57	(1.03–2.4)	0.04
Self-assessment tools/trackers	1.49	(1.1–2.0)	0.007

Each OR, odds ratio corresponds to a separate ordinal logistic regression analysis.

### Analysis 3: Clinical content themes associated with improved 12-month KCCQ-OS

The association between digital counseling content themes and 12-month KCCQ-OS was limited to the digital counseling modalities of videos and interactive tools/trackers during sessions 1–16, due to the above findings. The clinical theme that focused on MI to improve patient readiness for change (sessions 5–8) was associated with higher 12-month KCCQ-OS tertile scores when patients utilized tools/trackers (*P* = 0.04). Within the program segment that focused on CBT guidelines for CHF self-care behaviors (sessions 9–14) patient logon time was associated with higher 12-month KCCQ tertile scores on digital pages that presented multimedia videos (*P* = 0.02), as well as tools/trackers (*P* = 0.02). In sessions that enabled patients to review the connection between their HRQL and CHF self-care maintenance (sessions 15–16), logon time was associated with higher 12-month KCCQ-OS tertile scores (*P* = 0.01) – Table 4 and Supplementary Table 3 for details.

**TABLE 4:** Association between logon time for clinical content themes in sessions 1–16 and 12-month KCCQ-OS tertile.

Session	Clinical theme	Modality of content delivery	OR	95% CI	*P*-value
Session 5–8	Motivational interviewing	Self-assessment tools/trackers	39.9	(1.1–1413.0)	0.04
Session 9–14	CBT guide for CHF self-care	Videos: Expert guide and dramatic vignettes	464.7	(3.2–66778)	0.02
		Self-assessment tools/trackers	108.7	(2.1–5493.9)	0.02
Sessions 15–16	HRQL and self-care maintenance	Self-assessment tools/trackers	5.69	(1.5–22.2)	0.01

Each OR, odds ratio corresponds to a separate ordinal logistic regression analysis. CBT, cognitive behavioral therapy; CHF, chronic heart failure; HRQL, health-related quality of life.

## Discussion

The objective of this study was to identify therapeutic components of the digital counseling arm of the CHF-CePPORT trial that were positively associated with the 12-month KCCQ-OS endpoint (17). CHF-CePPORT used core components of behavioral counseling from CBT and MI that were delivered through three digital modalities: interactive tools/trackers, videos, and information pages. Due to the clinical organization of CHF-CePPORT, we were able to readily identify therapeutic components over the 12-month period of the intervention. The initial 4-month segment of CHF-CePPORT (sessions 1–16) was associated with improved HRQL. During this period participants were sent a digital link to the intervention on a weekly basis, which may have reinforced a sustained pattern of engagement with digital counseling resources.

The median number of total sessions that patients accessed in CHF-CePPORT was 17 (61% of the full protocol of 28 sessions). Therefore, it appears that patient engagement in the initial 16 sessions accounted for most of the therapeutic effect in CHF-CePPORT. This level of patient engagement has been observed in other trials of digital health as a threshold that is associated with improved clinical outcomes (11). The present study provides a more granular analysis of this therapeutic effect. Patient logon time with core components of behavioral counseling delivered through interactive tools/trackers and videos was associated with higher HRQL, but this outcome was not observed for patient engagement with digital information pages (comprised of narrative script and illustrations). This finding raises a potential concern since conventional patient education websites for CHF are largely comprised of digital pages that are filled with narrative scripts and illustrations.

The present results are also consistent with findings from a systematic review, in which patient adherence to CHF self-care behavior was significantly enhanced with the use of video interventions (20). In CHF-CePPORT, the videos were designed to engage patients more holistically with dramatic vignettes, expert summaries of self-help tips, and peer discussion about self-care. It remains to be determined whether the therapeutic effect of these videos was attributable to features such as positive role modeling, comments from health professionals that validated patient efforts at lifestyle change, or the dynamic presentation of explicit guidelines for CHF self-care. With this, our study adds to the policy recommendations observed, by promoting evidence-based features within cognitive behavioral and motivational interviewing models of counseling (13).

A recent review of mobile health technologies for patients with CHF highlights the lack of sustained patient engagement with these interventions as a clinically challenging issue (21). This was supported by evidence from a separate systematic review of mobile health interventions for CHF, where program usage was observed to be consistently low, with some studies reporting attrition rates of 30–60% (22). Understanding the ways in which patients engage with digital health interventions over clinically meaningful time intervals is a priority for current research.

Some strategies have shown promise in improving both treatment efficacy and sustained patient engagement with digital interventions for health behavior change. An early meta-analytic study reported that outcomes were improved with dynamic tailoring that matched program goals with the participant’s reported priorities for behavior change across repeated assessments. Dynamic tailoring with iterative feedback to patients, evoked greater treatment effects that remained significant in outcome assessments beyond 12-months (23). Current task force statements on digital counseling have not included explicit guidelines for dynamic tailoring (24), due in part to the limited availability of evidence. Nevertheless, the application of tailoring strategies in digital health has a clear potential to ensure that protocols for counseling and patient education are grounded within patient-centered goals for improved health status and quality of life.

The use of digital tailoring strategies to enhance the efficacy and usability of digital health interventions may become more prevalent with the emerging role of machine learning (ML) models in precision care for CHF. Predictive modeling based on ML is well-suited to identify how components of digital counseling programs can interface effectively with patient preferences. These preferences may be shaped by background attributes (e.g., socioeconomic status, education level, or severity of medical condition), health literacy level, motivation and skill for learning self-care behavior, and quality of social support for sustaining a lifestyle characterized by CHF self-care (25). The method of analysis used in this secondary study of CHF-CePPORT could be enhanced with the use of ML modeling.

Consistent with previous taskforce statements (1), it may be possible to better standardize and replicate positive outcomes from digital counseling programs when a theoretical framework is specified. In a recent meta-analysis on digital health interventions to manage hypertension, only 25% of trials identified a behavioral counseling model in their protocol (26). Moreover, heterogeneity in trial outcomes was significantly reduced and the treatment outcome was significantly improved among digital programs that specified a behavioral counseling model.

### Study limitations

Findings of the present study were based on outcomes from the digital counseling arm of the CHF-CePPORT trial, which limits the generalizability of our results (15). The digital counseling protocol in CHF-CePPORT was organized according to a pre-set sequence for scheduling patient access to program components, and this feature may differentiate the present digital counseling protocol from other digital programs of CHF self-care. Additionally, our analysis of patient usage of the various components of the CHF-CePPORT trial was limited to the segments of the trial (months 1–4, 5–8, and 9–12), the type of delivery (videos vs. tools/trackers vs. information pages), and the clinical themes. We were unable to provide a granular analysis of the specific types of videos (dramatic vignettes, expert summaries of self-help tips, peer discussion on self-care behavior and quality of life), interactive tools and trackers, or information pages that were utilized by patients in the digital counseling group. As reported in the primary outcome paper for CHF-CePPORT (15), enrolled patients presented with elevated baseline scores for the KCCQ-OS. Due to this ceiling effect, the primary analysis of CHF-CePPORT was not able to properly test whether the program was able to improve KCCQ outcomes over 12 months. Therefore, the primary outcome for CHF-CePPORT was a null finding. However, the follow-up analyses showed that the association between usage and 12-month improvement was significant for the treatment group but there was no association for the control group. This helps us to understand more clearly the potential therapeutic components for the intervention which are advisable to incorporate into subsequent trials. Furthermore, therapeutic components of digital counseling that were identified in this study may not be fully applicable to a sample of CHF patients that have greater impairment in health status. In addition, our sample had a positive balance between males and females; however, it was primarily Caucasian, and it did not include a large representation of individuals with low income. Further, education was elevated to a post-secondary level, which also affects the generalizability of our findings.

## Conclusion

This sub-study of the CHF-CePPORT trial was conducted to specify therapeutic components of an automated digital counseling program for CHF self-care. Increased KCCQ-OS at 12 months was associated with logon time in the initial 4 months for videos and interactive tools/trackers that delivered key components of CBT and MI. These results confirm the importance of using evidence-based models of behavioral counseling to promote CHF self-care and HRQL in a digital counseling program. In sum, the present results highlight the need to develop a sophisticated analytic strategy (e.g., with ML modeling) to identify therapeutic components of digital counseling, and in turn improve the standardization and replicability of digital interventions for CHF.

## Data availability statement

The datasets presented in this article are not readily available because the data are available from the corresponding author pending approval of Research Ethics Boards of participating institutions and on reasonable request received from qualified researchers trained in human subject confidentiality protocols. Requests to access the datasets should be directed to RN.

## Ethics statement

The studies involving human participants were reviewed and approved by the Research Ethics Board, University Health Network. The patients/participants provided their written informed consent to participate in this study.

## Author contributions

GF and SS: conceptualization, methodology, formal analysis, and writing – original draft, review, and editing. RN: conceptualization, methodology, formal analysis, writing – original draft, review, and editing, investigation (data contributed from the CHF-CePPORT trial), and supervision. All authors contributed to the article and approved the submitted version.
