# Supplementary material for: Therapeutic components of digital counseling for chronic heart failure
Source: Front Psychiatry. 2022 Oct 20;13:888524. doi: 10.3389/fpsyt.2022.888524 (PMC9631313; doi:10.3389/fpsyt.2022.888524)
Supplement: Supplementary file 1 [file Data_Sheet_1.docx]

**SUPPLEMENTARY DATA TABLE S1.**

**Ordinal Logistic Regression for the Association Between 12-month KCCQ-OS Tertile and Logon Hours for CHF-CePPORT Program Segments**

|  | **OR** | **95% CI** | **p-value** |
| --- | --- | --- | --- |
| 1A. Total Logon Hours for Months 1-4 (Session 1-16): | 1.31 | (1.1-1.5) | 0.001 |
| Age | 0.96 | (0.9-1.0) | 0.13 |
| Baseline LVEF |  |  |  |
| <35: reference |  |  |  |
| 35-40 | 0.9 | (0.3-3.1) | 0.91 |
| 41-45 | 10.8 | (2.2-51.8) | 0.003 |
| Baseline KCCQ-OS tertile |  |  |  |
| Median=58.5 (range, ≤ 74): reference |  |  |  |
| Median=82.9 (range, 74.1-90.4) | 22.2 | (5.3-92.7) | <0.001 |
| Median=95.2 (range, 90.5-100) | 3099.1 | (295.7-32474.1) | <0.001 |
| 1B. Total Logon Hours for Months 5-8 (Sessions 17-24) | 1.26 | (0.9-1.7) | 0.13 |
| Age | 0.98 | (0.9-1.0) | 0.32 |
| Baseline LVEF |  |  |  |
| <35: reference |  |  |  |
| 35-40 | 1.1 | (0.3-3.4) | 0.93 |
| 41-45 | 7.2 | (1.7-30.9) | 0.008 |
| Baseline KCCQ-OS tertile |  |  |  |
| Median=58.5 (range, ≤ 74): reference |  |  |  |
| Median=82.9 (range, 74.1-90.4) | 19.5 | (5.02-76.0) | <0.001 |
| Median=95.2 (range, 90.5-100) | 1403.7 | (177.7-11086.1) | <0.001 |
| 1C. Total Logon Hours for Months 9-12 (Sessions 25-28) | 1.42 | (0.8-2.7) | 0.28 |
| Age | 0.98 | (0.9-1.0) | 0.31 |
| Baseline LVEF |  |  |  |
| <35: reference |  |  |  |
| 35-40 | 0.98 | (0.3-3.2) | 0.98 |
| 41-45 | 6.5 | (1.6-27.0) | 0.01 |
| Baseline KCCQ-OS tertile |  |  |  |
| Median=58.5 (range, ≤ 74): reference |  |  |  |
| Median=82.9 (range, 74.1-90.4) | 17.8 | (4.7-67.6) | <0.001 |
| Median=95.2 (range, 90.5-100) | 1203.6 | (161.6-8967.0) | <0.001 |

*KCCQ-OS, Kansas City Cardiomyopathy Questionnaire – Overall Summary; LVEF, left ventricular ejection fraction; OR, odds ratio*

**SUPPLEMENTARY DATA TABLE S2.**

**Ordinal Logistic Regression for the Association Between 12-month KCCQ-OS Tertile and Logon Hours for Digital Counseling Modalities in Sessions 1-16**

|  | **OR** | **95% CI** | **p-value** |
| --- | --- | --- | --- |
| 2A. Total Logon Hours for Information/Education Pages | 1.09 | (0.5-2.2) | 0.80 |
| Age | 0.98 | (0.9-1.0) | 0.46 |
| Baseline LVEF |  |  |  |
| <35: reference |  |  |  |
| 35-40 | 1.1 | (0.3-3.6) | 0.88 |
| 41-45 | 5.9 | (1.4-23.8) | 0.013 |
| Baseline KCCQ-OS tertile |  |  |  |
| Median=58.5 (range, ≤ 74): reference |  |  |  |
| Median=82.9 (range, 74.1-90.4) | 18.4 | (4.9-69.5) | <0.001 |
| Median=95.2 (range, 90.5-100) | 957.6 | (137.7-6661.2) | <0.001 |
| 2B. Total Logon Hours for Videos: Expert Guideline, Dramatic Vignettes, and Peer Discussion | 1.57 | (1.0-2.4) | 0.04 |
| Age | 0.97 | (0.9-1.0) | 0.25 |
| Baseline LVEF |  |  |  |
| <35: reference |  |  |  |
| 35-40 | 1.0 | (0.3-3.3) | 0.99 |
| 41-45 | 7.1 | (1.7-30.0) | 0.007 |
| Baseline KCCQ-OS tertile |  |  |  |
| Median=58.5 (range, ≤ 74): reference |  |  |  |
| Median=82.9 (range, 74.1-90.4) | 17.1 | (4.4-66.0) | <0.001 |
| Median=95.2 (range, 90.5-100) | 1230.7 | (159.4-9503.6) | <0.001 |
| 2C. Total Logon Hours for Self-Assessment Tools/Trackers | 1.49 | (1.1-2.0) | 0.007 |
| Age | 0.97 | (0.9-1.0) | 0.19 |
| Baseline LVEF |  |  |  |
| <35: reference |  |  |  |
| 35-40 | 1.1 | (0.3-3.7) | 0.85 |
| 41-45 | 11.1 | (2.3-54.3) | 0.003 |
| Baseline KCCQ-OS tertile |  |  |  |
| Median=58.5 (range, ≤ 74): reference |  |  |  |
| Median=82.9 (range, 74.1-90.4) | 22.3 | (5.4-92.5) | <0.001 |
| Median=95.2 (range, 90.5-100) | 3133.4 | (290.7-33770.5) | <0.001 |
| *KCCQ-OS, Kansas City Cardiomyopathy Questionnaire – Overall Summary; LVEF, left ventricular ejection fraction; OR, odds ratio* | | | |

| **SUPPLEMENTARY DATA TABLE S3.**  **Ordinal Logistic Regression for the Association Between 12-month KCCQ-OS Tertile and Logon Hours for Clinical Themes of Digital Counseling in Sessions 1-16** |
| --- |

|  | **OR** | **95% CI** | **p-value** |
| --- | --- | --- | --- |
| 3A. Total Logon Hours for **Introduction** (Sessions 1-4) Using *Videos* | 4.9 | (0.05-517.3) | 0.50 |
| Age | 0.98 | (0.9-1.0) | 0.39 |
| Baseline LVEF |  |  |  |
| <35: reference |  |  |  |
| 35-40 | 1.1 | (0.3-3.5) | 0.90 |
| 41-45 | 5.7 | (1.4-22.9) | 0.01 |
| Baseline KCCQ-OS tertile |  |  |  |
| Median=58.5 (range, ≤ 74): reference |  |  |  |
| Median=82.9 (range, 74.1-90.4) | 17.7 | (4.7-67.1) | <0.001 |
| Median=95.2 (range, 90.5-100) | 964.04 | (138.7-6702.5) | <0.001 |
| 3B. Total Logon Hours for **MI** (Sessions 5-8) Using *Videos* | 47.3 | (0.1-15678.1) | 0.19 |
| Age | 0.98 | (0.9-1.0) | 0.48 |
| Baseline LVEF |  |  |  |
| <35: reference |  |  |  |
| 35-40 | 1.1 | (0.3-3.5) | 0.91 |
| 41-45 | 6.99 | (1.7-29.04) | 0.007 |
| Baseline KCCQ-OS tertile |  |  |  |
| Median=58.5 (range, ≤ 74): reference |  |  |  |
| Median=82.9 (range, 74.1-90.4) | 18.2 | (4.8-69.7) | <0.001 |
| Median=95.2 (range, 90.5-100) | 996.8 | (140.5-7073.2) | <0.001 |
| 3C. Total Logon Hours for **CBT Guidelines for CHF Self-Care** (Sessions 9-14) Using *Videos* | 464.7 | (3.2-66778.2) | 0.02 |
| Age | 0.98 | (0.9-1.0) | 0.52 |
| Baseline LVEF |  |  |  |
| <35: reference |  |  |  |
| 35-40 | 1.13 | (0.3-3.9) | 0.85 |
| 41-45 | 7.2 | (1.7-30.8) | 0.008 |
| Baseline KCCQ-OS tertile |  |  |  |
| Median=58.5 (range, ≤ 74): reference |  |  |  |
| Median=82.9 (range, 74.1-90.4) | 25.0 | (5.6-112.0) | <0.001 |
| Median=95.2 (range, 90.5-100) | 2018.8 | (212.98-19135.8) | <0.001 |
| 3D. Total Logon Hours for **HRQL and Self-Care Maintenance** (Session 15-16) Using *Videos* | 3.2 | (0.8-12.8) | 0.10 |
| Age | 0.99 | (0.9-1.0) | 0.65 |
| Baseline LVEF |  |  |  |
| <35: reference |  |  |  |
| 35-40 | 1.4 | (0.4-4.6) | 0.62 |
| 41-45 | 8.7 | (1.97-38.8) | 0.004 |
| Baseline KCCQ-OS tertile |  |  |  |
| Median=58.5 (range, ≤ 74): reference |  |  |  |
| Median=82.9 (range, 74.1-90.4) | 26.8 | (5.8-122.8) | <0.001 |
| Median=95.2 (range, 90.5-100) | 1553.1 | (181.1-13320.2) | <0.001 |
| 3E. Total Logon Hours for **Introduction** (Sessions 1-4) Using *Tools/Trackers* | 2.9 | (0.2-51.0) | 0.47 |
| Age | 0.98 | (0.9-1.0) | 0.42 |
| Baseline LVEF |  |  |  |
| <35: reference |  |  |  |
| 35-40 | 1.0 | (0.3-3.4) | 0.99 |
| 41-45 | 6.3 | (1.5-25.8) | 0.01 |
| Baseline KCCQ-OS tertile |  |  |  |
| Median=58.5 (range, ≤ 74): reference |  |  |  |
| Median=82.9 (range, 74.1-90.4) | 19.0 | (5.0-72.8) | <0.001 |
| Median=95.2 (range, 90.5-100) | 1118.0 | (149.0-8391.3) | <0.001 |
| 3F. Total Logon Hours for **MI** (Sessions 5-8) Using *Tools/Trackers* | 39.9 | (1.1-1413.0) | 0.04 |
| Age | 0.98 | (0.9-1.0) | 0.37 |
| Baseline LVEF |  |  |  |
| <35: reference |  |  |  |
| 35-40 | 1.2 | (0.4-3.9) | 0.77 |
| 41-45 | 8.2 | (1.9-36.1) | 0.005 |
| Baseline KCCQ-OS tertile |  |  |  |
| Median=58.5 (range, ≤ 74): reference |  |  |  |
| Median=82.9 (range, 74.1-90.4) | 22.8 | (5.6-93.3) | <0.001 |
| Median=95.2 (range, 90.5-100) | 1899.8 | (207.6-17386.4) | <0.001 |
| 3G. Total Logon Hours for **CBT Guidelines for CHF Self-Care** (Sessions 9-14) Using *Tools/Trackers* | 108.7 | (2.1-5493.9) | 0.02 |
| Age | 0.99 | (0.9-1.0) | 0.56 |
| Baseline LVEF |  |  |  |
| <35: reference |  |  |  |
| 35-40 | 1.5 | (0.4-5.2) | 0.56 |
| 41-45 | 9.4 | (2.0-43.7) | 0.004 |
| Baseline KCCQ-OS tertile |  |  |  |
| Median=58.5 (range, ≤ 74): reference |  |  |  |
| Median=82.9 (range, 74.1-90.4) | 29.8 | (6.2-143.6) | <0.001 |
| Median=95.2 (range, 90.5-100) | 3138.4 | (281.8-34946.7) | <0.001 |
| 3H. Total Logon Hours for **HRQL and Self-Care Maintenance** (Session 15-16) Using *Tools/Trackers* | 5.69 | (1.5-22.2) | 0.01 |
| Age | 0.98 | (0.9-1.0) | 0.38 |
| Baseline LVEF |  |  |  |
| <35: reference |  |  |  |
| 35-40 | 1.5 | (0.4-5.0) | 0.55 |
| 41-45 | 12.0 | (2.3-61.5) | 0.003 |
| Baseline KCCQ-OS tertile |  |  |  |
| Median=58.5 (range, ≤ 74): reference |  |  |  |
| Median=82.9 (range, 74.1-90.4) | 30.7 | (6.3-149.5) | <0.001 |
| Median=95.2 (range, 90.5-100) | 3341.4 | (279.0-40014.6) | <0.001 |

*CBT, cognitive behavioral therapy; HRQL, health-related quality of life; KCCQ-OS, Kansas City Cardiomyopathy Questionnaire – Overall Summary; LVEF, left ventricular ejection fraction; MI, motivational interviewing; OR, odds ratio*

**SUPPLEMENTARY DATA TABLE S4.**

**Ordinal Logistic Regression for the Association Between Logon Hours During Sessions 1-16 and 12-Month KCCQ Tertiles of Subscales: Clinical Summary, Social Limitation, and Total Symptoms**

|  | **OR** | **95% CI** | **p-value** |
| --- | --- | --- | --- |
| **4A. KCCQ Clinical Summary Subscale** |  |  |  |
| Total Logon Hours for Sessions 1-16 | 1.02 | (0.9-1.2) | 0.76 |
| Age | 0.98 | (0.9-1.0) | 0.35 |
| Baseline LVEF |  |  |  |
| <35: reference |  |  |  |
| 35-40 | 0.76 | (0.3-2.3) | 0.62 |
| 41-45 | 1.72 | (0.5-6.3) | 0.41 |
| Baseline KCCQ-CS tertile |  |  |  |
| Median=58.5 (range, ≤ 74): reference |  |  |  |
| Median=82.9 (range, 74.1-90.4) | 21.3 | (6.2-73.8) | <0.001 |
| Median=95.2 (range, 90.5-100) | 430.4 | (82.3-2252.1) | <0.001 |
| **4B. KCCQ Social Limitation Subscale** |  |  |  |
| Total Logon Hours for Sessions 1-16 | 1.15 | (1.0-1.3) | 0.05 |
| Age | 0.96 | (0.9-1.0) | 0.07 |
| Baseline LVEF |  |  |  |
| <35: reference |  |  |  |
| 35-40 | 0.57 | (0.2-1.7) | 0.32 |
| 41-45 | 3.23 | (0.9-12.1) | 0.08 |
| Baseline KCCQ-SL tertile |  |  |  |
| Median=58.5 (range, ≤ 74): reference |  |  |  |
| Median=82.9 (range, 74.1-90.4) | 17.2 | (5.1-57.6) | <0.001 |
| Median=95.2 (range, 90.5-100) | 388.4 | (70.9-2128.5) | <0.001 |
| **4C. KCCQ Total Symptoms Subscale** |  |  |  |
| Total Logon Hours for Sessions 1-16 | 1.14 | (1.0-1.3) | 0.05 |
| Age | 0.99 | (0.95-1.02) | 0.48 |
| Baseline LVEF |  |  |  |
| <35: reference |  |  |  |
| 35-40 | 1.16 | (0.4-3.1) | 0.78 |
| 41-45 | 1.98 | (0.6-6.1) | 0.24 |
| Baseline KCCQ-TS tertile |  |  |  |
| Median=58.5 (range, ≤ 74): reference |  |  |  |
| Median=82.9 (range, 74.1-90.4) | 10.8 | (3.5-33.3) | <0.001 |
| Median=95.2 (range, 90.5-100) | 67.3 | (18.0-251.4) | <0.001 |

*KCCQ-CS, Kansas City Cardiomyopathy Questionnaire – Clinical Summary; KCCQ-SL, Kansas City Cardiomyopathy Questionnaire – Social Limitation; KCCQ-TS, Kansas City Cardiomyopathy Questionnaire – Total Symptoms; LVEF, left ventricular ejection fraction; MI, motivational interviewing; OR, odds ratio*
